# Feasibility and usability of GPS data in exploring associations between training load and running-related knee injuries in recreational runners

**DOI:** 10.1186/s13102-022-00472-8

**Published:** 2022-04-28

**Authors:** Kyra L. A. Cloosterman, Tryntsje Fokkema, Robert-Jan de Vos, Ben van Oeveren, Sita M. A. Bierma-Zeinstra, Marienke van Middelkoop

**Affiliations:** 1 Department of General Practice, Erasmus MC University Medical Centre, P.O. Box 2040, 3000 CA Rotterdam, The Netherlands; 2 Department of General Practice and Elderly Care Medicine, University Medical Centre Groningen, University of Groningen, Groningen, The Netherlands; 3 Department of Orthopaedics and Sports Medicine, Erasmus MC University Medical Centre, Rotterdam, The Netherlands; 4 Move-Metrics, Ede, The Netherlands

**Keywords:** Running, Sports injuries, Knee complaints, Athletes, ACWR

## Abstract

**Background:**

The purpose of the present study was to explore the feasibility of collecting GPS data and the usability of GPS data to evaluate associations between the training load and onset of running-related knee injuries (RRKIs).

**Methods:**

Participants of the INSPIRE-trial, a randomized-controlled trial on running injury prevention, were asked to participate in this study. At baseline, demographic variables were collected. Follow-up questionnaires assessed information on RRKIs. Participants with a new reported RRKI and uninjured participants were sent a GPS export request. Weekly GPS-based training distances were used to calculate Acute:Chronic Workload Ratios (ACWRs).

**Results:**

A total of 240 participants (62.7%) tracked their running training sessions with the use of a GPS-enabled device or platform and were willing to share their GPS data. From the participants (N = 144) who received a GPS export request, 50.0% successfully shared their data. The majority (69.4%) of the shared GPS data were usable for analyses (N = 50). GPS data were used to present weekly ACWRs of participants with and without an RRKI eight weeks prior to RRKI onset or running event.

**Conclusions:**

It seems feasible to collect GPS data from GPS-enabled devices and platforms used by recreational runners. The results indicate that GPS data is usable to calculate weekly ACWRs to evaluate associations between training load and onset of RRKIs in recreational runners. Therefore, GPS-based ACWR measures can be used for future studies to evaluate associations between training load and onset of RRIs.

**Supplementary Information:**

The online version contains supplementary material available at 10.1186/s13102-022-00472-8.

## Introduction

Running is one of the most popular forms of physical exercise and is associated with positive effects on a range of health benefits [1]. However, a high number of runners experience a running-related injury (RRI) [2–4]. A recent systematic review of Kakouris et al. showed an overall injury incidence of 40.2% [5]. Up to 80% of all RRIs are thought to develop as a consequence of overuse [6]. Overuse injuries can occur when the training load exceeds the runners’ load capacity for adaptive tissue repair [7, 8]. Therefore, it is believed that the onset and development of RRIs is strongly related to an imbalance between training and recovery [8–10]. However, the estimation of load capacity is difficult since the training load varies due to variation within and between individuals [10, 11].

The Acute:Chronic Workload Ratio (ACWR) can be used to calculate changes in training load and is calculated by dividing the acute training load (e.g. covered distance in the past week) by the chronic workload (e.g. covered distance in the past four weeks) [12, 13]. Research within team sport populations, like football, soccer and rugby, reported that a ratio of 1.5 or higher is associated with increased injury risk compared to a ratio of 0.8 to 1.3 [12, 14]. One study investigated the relationship between ACWR and injury risk in a mixed endurance sports population [15]. The authors concluded that endurance athletes could minimize their injury risk by maintaining moderate to high chronic training loads while avoiding high spikes in acute training load. Furthermore, a recent study in competitive runners showed that a fortnightly low increase of the ACWR (0.10–0.78) is related to an increased risk of sustaining an injury [16]. Last, a recent study in competitive trail runners reported a significant weekly increase in ACWR for session-rate of perceived exertion, total distance and training time in the weeks prior to an injury’s occurrence [17].

Questionnaires used to determine training characteristics retrospectively are reported to include inaccuracy due to recall bias [18]. Therefore, the use of global positioning systems (GPS) data to collect training information may be a more accurate method. Nowadays, more than 75% of runners use GPS-enabled devices, like sports watches, smartphone applications and activity trackers, to track their training activities [19, 20]. These devices can accurately measure several aspects of training load, such as distance and speed [20–22]. However, little is known about the feasibility of collecting GPS data and the usability of GPS data to study the ACWR to assess injury risk in runners. Examining injury risk is especially important in runners with a running-related knee injury (RRKI), since this is the most commonly reported injury in runners with an incidence of 26.2% in non-ultramarathoners and up to one third of the runners with an RRKI still experience complaints after one year [2, 5, 23]. Therefore, the aims of this study were to 1) explore the feasibility of collecting GPS data from recreational runners and 2) examine the usability of GPS data to evaluate associations between training load and onset of RRKIs with the use of ACWR.

## Materials and methods

### Study design

This study is part of the INtervention Study on Prevention of Injuries in Runners (INSPIRE) trial, a randomized-controlled trial to investigate the effect of an online injury prevention program on the number of RRIs [24]. After completing the baseline questionnaire, follow-up questionnaires were sent (i) two weeks before the running event; (ii) one day after the running event; (iii) four weeks after the running event. Participants with a new RRKI during follow-up were sent an additional knee-specific follow-up questionnaire at an average of 16 months (range 11.7–18.6) after baseline. GPS usage questions were sent at an average of 20 months (range 15.8–23.0) after baseline. The INSPIRE trial (trial registration number NTR5998) was funded by the Netherlands Organization for Health Research and Development (ZonMW, grant number 536001001). Medical ethics approval was obtained by the Medical Ethical Committee of the Erasmus Medical Centre Rotterdam, The Netherlands (MEC-2016-292).

### Participants

Runners who registered for one of three selected running events (distances 5.0–42.2 kilometer (km)) in 2017 were invited to participate in this study. Interested runners, aged 18 years or older, were asked to provide informed consent and to fill in the baseline questionnaire. For the current study purpose, participants with a new RRKI during follow-up and participants without an RRI in the past and during follow-up were included. Exclusion criteria were no knowledge of the Dutch language and no access to internet or e-mail. For the current study purpose, participants were excluded if they did not train with the use of a GPS-enabled device or platform or if they were not willing to share their GPS data. Furthermore, exclusion criteria were the use of a GPS-enabled device or platform without option to export GPS data and the estimated use of a device or platform in less than 80% of training sessions. Participants without an RRI were excluded if they reported a new RRI between baseline and the GPS usage questions.

### Questionnaires and procedures

**Fig. 1:**
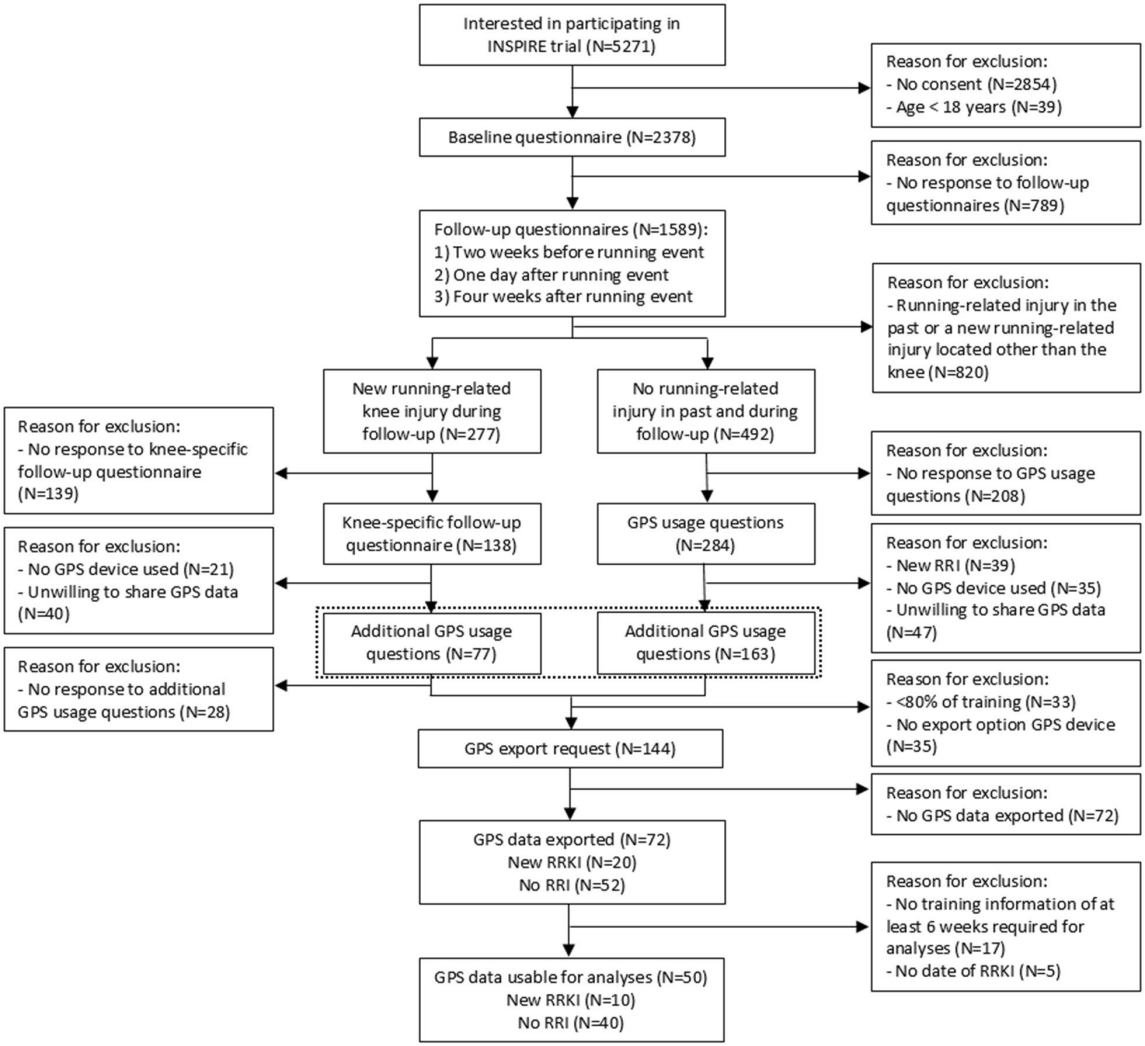
Flowchart of the participants

A flowchart of the procedures is presented in Fig. 1. In the baseline questionnaire, information on demographics (sex, age weight and height) and training characteristics (average weekly training frequency, hours, distance (km) and running speed (minutes per km) over the previous three months) were collected. Furthermore, information on running experience (years), RRI in the 12 months before baseline (yes/no) and distance of registered running event were obtained. In the follow-up questionnaires, participants were asked if they sustained a new RRI since completing the previous questionnaire (yes/no). If yes, location of RRI and number of weeks the participant suffered from the RRI were collected. In the knee-specific follow-up questionnaire, participants were asked if they tracked their training sessions with a GPS-enabled device or platform (yes/no) and if yes, if they were willing to share these data. The same questions were asked to the participants without an RRI (Fig. 1). Furthermore, these participants were asked if they suffered a new RRI since completing the last follow-up questionnaire (yes/no). Next, additional GPS usage questions were sent to participants who were willing to share GPS data. The additional GPS usage questions collected information on the brand of GPS-enabled device or platform, the number of recorded training sessions (< 80% or 80–100% of all sessions) and training type (all training sessions, endurance training, tempo training and interval training). A GPS export request was sent to the participants who met the inclusion criteria for the current study purpose. Manuals on how to share GPS data with the researchers were provided for the most popular platforms (i.e. Garmin, Strava, TomTom, Runkeeper and Runtastic). In order of the researchers, Move-Metrics, a company specialized in data analysis for sport and health, standardized the different activity file formats (.tcx,.fit,.json,.gpx) and derived descriptives from the activity files required for this study [25]. Details on characteristics measured with GPS are outlined in Additional file 1: Appendix A.

### Measurements

Body Mass Index (BMI) was calculated using weight and height. Based on the registered distances of the running event, participants were categorized into a short-distance group (i.e. 5–10.55 km) and long-distance group (i.e. 21.1–42.2 km) for comparison. If participants reported that they suffered an RRKI, the date of RRKI onset was calculated using the date of completion of the questionnaire minus the number of weeks a participant already suffered from the RRKI. If the date of RRKI onset could not be calculated within two weeks of certainty due to missing values, participants were excluded.

GPS data of the 11 weeks before the onset of the RRKI were selected to evaluate the associations between training load and the onset of RRKIs. We chose the 11 weeks of training load based on a combination of clinical experience and availability of data. For participants without an RRI, GPS data of the 11 weeks before the running event were selected. GPS data were divided into weekly blocks from Monday to Sunday and usable for analyses if it contained training information of at least six out of 11 weeks required for analyses. With the use of the GPS data, the average training minutes, distance (km) and running speed (minutes per km) were calculated for every training session. For each week, training loads were calculated as the total sum of the training distance. If a participant did not perform a training session for a week, a value of zero was included. Weekly ACWRs were calculated with the use of the coupled rolling average model in which acute training load (average training load of the present week) is divided by the chronic load (average training load of the present week and previous three weeks) [13].

### Outcomes

The primary outcome measure of this study was the feasibility of collecting GPS data in terms of the percentage of participants who were willing to share GPS data and the percentage of collected GPS data useable for analyses. The secondary outcome measure was the usability of GPS data to determine the weekly ACWR of participants with and without an RRKI. An RRKI was defined as any self-reported musculoskeletal complaint of the knee due to running activities, which restricted the amount of running (distance, duration, speed or frequency) for at least one week or needed medical consultation [2, 3, 26].

### Statistical analyses

Normality of the data was checked statistically using the Shapiro–Wilk test. Descriptive statistics were used to describe baseline characteristics, expressed in frequency or average and standard deviations (SDs). Baseline characteristics between participants who did and did not share GPS data were compared using independent sample t-tests (normally distributed continuous data), Mann–Whitney U tests (not normally distributed continuous data) and chi-square tests (dichotomous data). The same tests were used to explore differences in baseline characteristics between participants with an RRKI and without an RRI who shared GPS data usable for analyses. With the use of GPS data, training characteristics and corresponding SDs were calculated and compared between participants with an RRKI and without an RRI using independent sample t-tests. The same tests were used to examine differences between the long-distance and short-distance group. P-values < 0.05 were regarded as statistically significant. All analyses were performed using SPSS version 25.0 (SPSS Inc, Chicago, Illinois).

## Results

In total, 2378 runners participated in the INSPIRE trial (Fig. 1). On one hand, 277 (14.4%) participants reported an RRKI during follow-up and received an additional knee-specific follow-up questionnaire. A total of 138 (49.8%) participants responded to this additional questionnaire. Of those, 117 participants (84.8%) reported the use of a GPS-enabled device or platform to track their training sessions, of which 77 participants (65.8%) were willing to share their GPS data and received additional GPS usage questions. A total of 49 (63.6%) participants responded to these additional questions. On the other hand, 492 participants without an RRI in the past and during follow-up received GPS usage questions. Of the 284 (57.7%) responders, 39 (13.7%) participants reported a new RRI and were therefore excluded. Of the remaining participants, 210 (85.7%) participants used a GPS-enabled device or platform, of which 163 (77.6%) participants were willing to share their GPS data and received additional GPS usage questions.

### Feasibility of GPS data collection

From both RRKI and RRI branches, a total of 212 participants responded to the additional GPS usage questions. Most reported GPS-enabled devices or platforms were Strava (30.2%) and Garmin (28.3%) (Table 1). Participants were excluded from receiving the GPS export request if they tracked less than 80% of their training sessions (N = 33) or if their GPS-enabled device or platform had no option to export GPS data (N = 35). Of the participants who received a GPS export request, 72 (50.0%) participants shared their GPS data. After receiving GPS data, 17 participants were excluded because their GPS data did not contain training information of at least six out of 11 weeks required for analyses and five participants were excluded because the date of RRKI onset could not be given within a certainty of two weeks. GPS data of a total of 50 (69.4%) participants were usable for analyses.

**Table 1:** GPS usage responses

	Responders to the additional GPS usage questions (N = 212)
GPS-enabled device or platform	
Strava	64 (30.2)
Garmin	60 (28.3)
TomTom	43 (20.3)
Runkeeper	33 (15.6)
Polar	30 (14.2)
Runtastic	8 (3.8)
Nike + running	10 (4.7)
Other	14 (6.6)
Use of ≥ 2 GPS-enabled devices and/or platforms	53 (25.0)
GPS used ≥ 80% of training sessions	179 (84.4)
All training sessions recorded^†^	167 (93.3)

Data are presented as N (%)

^†^Based on runners who tracked at least 80% of their training sessions

### Usability of GPS data to determine the training load

Compared to the participants (N = 72) who received the GPS export request but did not share GPS data, participants (N = 72) who shared GPS data had significantly more running experience (9.1 years vs. 8.3 years, p = 0.02) and trained more times a week (2.7 vs. 2.6, p = 0.01) (Additional file 1: Appendix B). Participants (N = 50) who shared GPS data usable for analyses were on average 44.9 (SD 11.6) years old and the majority registered for a long-distance running event (72.0%) (Table 2). No significant baseline differences between participants with an RRKI and without an RRI who shared GPS data usable for analyses were found (Table 2).

**Table 2:** Baseline characteristics of runners who shared GPS data usable for analyses

	Total (N = 50)	RRKI^‡^ (N = 10)	No RRI^§^ (N = 40)
**Demographic characteristics**			
Sex (male)	36 (72.0)	8 (80.0)	28 (70.0)
Age (years)^†^	44.9 (11.6)	49.7 (12.6)	43.7 (11.2)
BMI (kg/m^2^)^†¶^	23.2 (2.1)	23.3 (1.1)	23.2 (2.3)
**Training characteristics**			
Running experience (years)^†^	9.2 (10.5)	14.3 (16.8)	7.9 (8.0)
Weekly training frequency^†^	2.5 (0.9)	2.3 (0.8)	2.6 (1.0)
Weekly training hours^†^	2.8 (1.5)	2.6 (1.3)	2.9 (1.5)
Weekly training distance (km)^†^	25.0 (15.0)	21.6 (10.3)	25.9 (15.9)
Running speed (min/km)^†^	5.9 (0.9)	5.8 (0.7)	5.9 (1.0)
**Running event**			
Distance registered for			
Short-distance (5–10.55 km)	14 (28.0)	4 (40.0)	10 (25.0)
Long-distance (21.1–42.2 km)	36 (72.0)	6 (60.0)	30 (75.0)

Categorical data are presented as N (%) and continuous data (^†^) as average (SD). No statistically significant difference between participants who did and did not share GPS data usable for analyses

^‡^Running-related knee injury

^§^Running-related injury

^¶^Body Mass Index

Participants with an RRKI trained on average 51.1 (SD 26.2) minutes with a distance of 8.2 (SD 4.2) km per training in the eight weeks prior to the RRKI onset (Table 3). Participants with an RRKI in the long distance group trained significantly more often compared to participants without an RRI (3.5 vs. 2.9 times a week, p ≤ 0.01). Of the participants who registered for a short-distance running event, participants without an RRI trained at a significantly higher speed (5.3 vs. 5.6 min/km, p ≤ 0.01) compared to participants with an RRKI.

**Table 3:** Training characteristics measured by GPS eight weeks before onset of RRKI or running event

	Total		Short-distance event		Long-distance event	
	RRKI (N = 10)	No RRI (N = 40)	RRKI (N = 4)	No RRI (N = 10)	RRKI (N = 6)	No RRI (N = 30)
Weekly training frequency	3.2 (1.4)	2.9 (1.2)	2.8 (1.2)	2.7 (1.2)	3.5 (1.5)	2.9 (1.1)*
**Each training session**						
Average training duration (min)	51.1 (26.2)	56.0 (32.8)	36.7 (13.0)	33.1 (13.9)	60.4 (28.4)	63.3 (33.7)
Average training distance (km)	8.2 (4.2)	9.6 (5.3)	1.7 (10.8)	6.0 (2.4)	9.9 (4.5)	10.8 (5.4)
Average running speed (min/km)	6.0 (1.0)	5.6 (1.1)	5.6 (1.0)	5.3 (1.0)*	5.9 (1.0)	5.6 (1.1)

Data are presented as average (SD)

*Statistically significant difference between participants with an RRKI and participants with no RRI (p < 0.05)

**Fig. 2:**
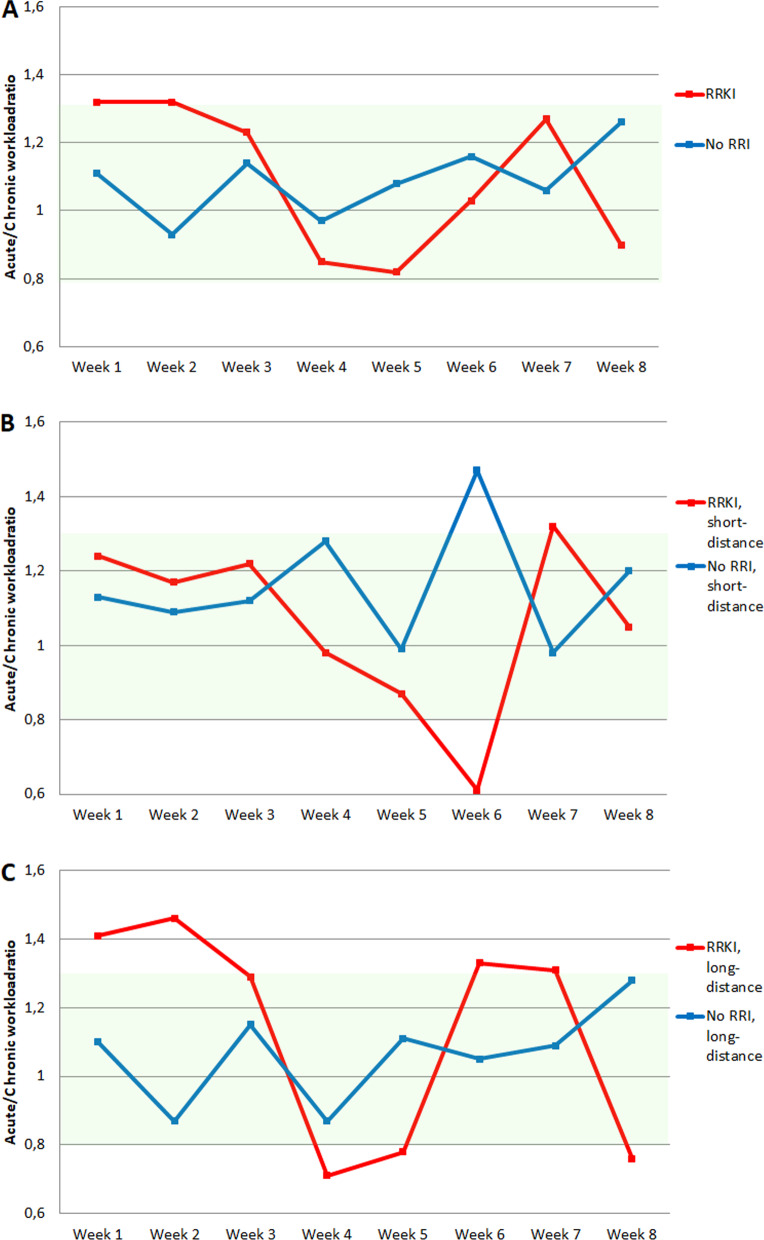
Weekly Acute:Chronic Workload ratios (ACWRs) of participants with a running-related knee injury (RRKI) and without a running-related injury (RRI). ACWRs are calculated by weekly training distance. **A** For participants with an RRKI, weekly ACWR is calculated for the eight weeks before onset of the RRKI. For participants without an RRI, weekly ACWR is calculated for the eight weeks before start of the running event. For both groups the ACWRs are also calculated based on registered distance of running event: **B** short-distance (5–10.55 km) and **C** long-distance (21.1–42.2 km). ACWRs within the range of 0.8 to 1.3 (“green zone”) were regarded as normal [12]

Figure 2 and Additional file 1: Appendix C present weekly ACWRs of participants with and without an RRKI eight weeks prior to RRKI onset or registered running-event. Weekly ACWRs of participants for a long-distance and short-distance running event are also presented. As observed in Fig. 2A, participants without RRI showed relative stable average values of ACWR over the eight-week period, while participants with an RRKI showed more fluctuated average values of ACWR. Participants with an RRKI who registered for a long-distance running event showed more fluctuated average values of ACWR compared to participants without an RRI (Fig. 2C).

## Discussion

Almost two third of the participants used a GPS-enabled device or platform to track their running training sessions and were willing to share GPS data. It therefore seems feasible to collect data from GPS-enabled devices and platforms that are used by recreational runners. However, caution is advised since only half of the participants who received a GPS export request did actually share their GPS data and of this data two third was usable for analyses. Our study showed that GPS data derived from GPS-enabled devices and platforms from recreational runners contained variables like training frequency, duration, distance, and speed and these can be used for calculation of ACWR values.

More than 85% of the participants used a GPS-enabled device or platform to track their running training sessions. This is comparable with previous studies in which more than 75% of runners used GPS-enabled devices [19, 20]. Since GPS data were collected from devices owned by the participant, no effort and costs were made for these measurements. Of the participants (N = 144) who received a GPS export request, 50% did not share GPS data. Possible reasons to not share GPS data are the need to perform multiple steps to share GPS data and concerns about privacy handling of data. Therefore, we expect that user-friendly sharing, with more information on privacy handling of data may improve data collection for research purposes. Nevertheless, this study shows that it seems feasible to collect GPS data from several brands of GPS-enabled devices and platforms used and owned by recreational runners.

Next, it is important that GPS data contain useful data to calculate training load. Our study showed that GPS data derived from GPS-enabled devices and platforms from recreational runners contained variables like training frequency, duration, distance, and speed. With the use of the variable training distance we calculated ACWRs. Furthermore, previous research reported that GPS-enabled devices can accurately measure this variable, perhaps even more accurate than questionnaires [20–22]. So GPS data derived from GPS-enabled devices offer a better alternative to calculate training load compared to questionnaires. However, there is much debate about the best model to calculate training load in runners. For many years, runners were advised to limit the increase in their weekly training load to < 10% in order to minimize the risk of injury [27]. Recently, studies concluded that the ACWR can be used to examine the relationship between training load and injury risk [12, 14]. The best model to calculate ACWR is unclear and some studies reported that the exponentially weighted moving average (EWMA) is a more sensitive model to detect associations between training load and injury [28]. So far, only a few studies examined associations between training load and injury risk in runners [15–17]. Future prospective studies with large sample sizes are necessary to determine the best method to calculate training load in recreational runners and to explore associations between training load and onset of RRIs.

### Strengths and limitations

A strength of the current study is that it is the first study that provides information on the feasibility of GPS data collection in recreational runners. Furthermore, this is the first study that explored the usability of GPS data to evaluate associations between training load and onset of RRKIs with the use of ACWRs. However, this study has a number of limitations. The loss-to-follow-up (51.2%) was relatively high. Reason for this high percentage might be the long follow-up of 20 months (range 15.8–23.0). Besides the knee-specific follow-up questionnaire, there was no contact with the participants between the follow-up questionnaires and the GPS usage questions. Due to this long follow-up, participants might have become less interested to answer this questionnaire, which was also not announced when this study started. The questionnaires were not validated. However, the definition of RRIs and data collection using this approach has been frequently applied and published in the past [29, 30]. Another limitation is the date of RRKI onset. Participants were asked to estimate the duration of their RRKI. Because only three follow-up questionnaires were sent to the participants, this may have caused recall bias. Furthermore, ACWRs of participants without an RRI were calculated based on the 11 weeks before the running event. For participants with a new reported RRKI, ACWRs were calculated based on the 11 weeks before RRKI onset. However, these weeks included at least five out of the 11 weeks before the running event and were therefore comparable to the weeks selected for participants without an RRI. A loss of training data in a specific period might have large impact on the ACWR in that period. This might have influenced the results, although we cannot estimate the impact of the missing data. The threshold to determine whether GPS data collection is feasible was not defined as it depends on multiple factors such as population heterogeneity and population size. Furthermore, due to the small sample sizes per subgroup, no statistical test was performed to compare ACWR values between participants with and without an RRKI. We described the use of GPS-enabled devices or platforms used by the participants. However, for some devices or platforms it was not clear if a sports watch or smartphone was used to track the running training. When using GPS data derived from different GPS-enabled devices or platforms, differences in training recording and device differences can be expected. Therefore, researchers should keep in mind potential bias to usage, device specifications and sensor-position.

In conclusion, this study shows that it seems feasible to collect training characteristics from GPS-enabled devices and platforms used by recreational runners. The results indicate that GPS data is usable to calculate weekly ACWRs to evaluate associations between training load and onset of RRKIs in recreational runners. Therefore, GPS-based ACWR measures can be used for future studies to evaluate associations between training load and onset of RRIs.

## Supplementary Information

**Additional file 1**. Supplementary material.

## Data Availability

The datasets used and/or analysed during the current study are available from the corresponding author on reasonable request.

## Abbreviations

RRI: Running-related injury
ACWR: Acute:Chronic Workload Ratio
GPS: Global positioning systems
RRKI: Running-related knee injury
INSPIRE: INtervention Study on Prevention of Injuries in Runners
Km: Kilometer
BMI: Body Mass Index
SD: Standard deviation
