# Supplementary material for: Feasibility and usability of GPS data in exploring associations between training load and running-related knee injuries in recreational runners
Source: BMC Sports Sci Med Rehabil. 2022 Apr 28;14:78. doi: 10.1186/s13102-022-00472-8 (PMC9052652; doi:10.1186/s13102-022-00472-8)
Supplement: Supplementary file 1 — Additional file 1. Supplementary material. [file 13102_2022_472_MOESM1_ESM.docx]

| Appendix A. Characteristics measured by GPS-enabled device or platform | | | | |
| --- | --- | --- | --- | --- |
|  | **GPS-enabled device** | | | |
|  | **Strava** | **Garmin** | **Runkeeper** | **TomTom** |
| Duration (sec) | Yes | Yes | Yes | Yes |
| Distance (meters) | Yes | Yes | Yes | Yes |
| Average speed (km/h) | Yes | Yes | Yes | Yes |
| Heart rate (bpm) | Product^†^ | Product | No | Product |
| Cadence (spm) | Product | Product | No | No |
| Altitude (meters) | Yes | Yes | Yes | Yes |
| Stance time (ms) | Product | Product | No | No |
| Vertical oscillation (cm) | Product | Product | No | No |
| Total steps | Product | No | No | No |
| ^†^ Characteristic measured depending on type of GPS-enabled device. | | | | |

| Appendix B. Baseline characteristics of participants who did and did not share GPS data after receiving the GPS export request | | | |
| --- | --- | --- | --- |
|  | **GPS data shared** | | |
|  | **Total (N=144)** | **Yes (N=72)** | **No (N=72)** |
| **Demographic characteristics** |  |  |  |
| Sex (male) | 91 (63.2) | 51 (70.8) | 40 (55.6) |
| Age (years)^†^ | 44.9 (12.6) | 44.5 (12.0) | 45.2 (13.2) |
| BMI (kg/m2)^†‡^ | 23.5 (2.9) | 23.1 (2.4) | 23.9 (3.3) |
| **Training characteristics** |  |  |  |
| Running experience^†^ | 8.7 (10.1) | 9.1 (10.6) | 8.3 (9.6)* |
| Weekly training frequency^†^ | 2.6 (1.3) | 2.7 (1.0) | 2.6 (1.4)* |
| Weekly training hours^†^ | 2.8 (1.4) | 2.9 (1.5) | 2.7 (1.3) |
| Weekly training distance (km)^†^ | 26.2 (19.3) | 25.3 (14.8) | 27.1 (22.9)* |
| Running speed (min/km)^†^ | 6.1 (1.1) | 5.9 (0.9) | 6.2 (1.3) |
| **Injuries** |  |  |  |
| RRI^§^ 12 months before baseline | 11 (7.6) | 8 (11.1) | 3 (4.2) |
| New RRKI^¶^ during follow-up | 35 (24.3) | 20 (27.8) | 15 (20.8) |
| **Running event** |  |  |  |
| Distance registered for: |  |  |  |
| Short-distance (5-10.55km) | 55 (38.2) | 22 (30.6) | 33 (45.8) |
| Long-distance (21.1-42.2km) | 89 (61.8) | 50 (69.4) | 39 (54.2) |
| Categorical data are presented as N (%) and continuous data (^†^) as average (SD). * = statistically significant difference between responders and non-responders (p<0.05); ^‡^ Body Mass Index;  ^§^ Running-related injury; ^¶^ Running-related knee injury. | | | |

| Appendix C. Weekly Acute:Chronic Workload Ratio (ACWR) calculated by weekly training distance in participants with an RRKI and without an RRI | | | | | | |
| --- | --- | --- | --- | --- | --- | --- |
| **ACWR** | **Total** | | **Short-distance event**^†^ | | **Long-distance event**^†^ | |
|  | RRKI^‡^ | No RRI^§^ | RRKI | No RRI | RRKI | No RRI |
|  | (N=10) | (N=40) | (N=4) | (N=10) | (N=6) | (N=30) |
| Week 1 | 1.34 (1.1) | 1.11 (0.5) | 1.24 (0.8) | 1.13 (0.5) | 1.41 (1.4) | 1.10 (0.5) |
| Week 2 | 1.35 (0.4) | 0.93 (0.7) | 1.17 (0.3) | 1.09 (1.1) | 1.46 (0.5) | 0.87 (0.5) |
| Week 3 | 1.26 (0.4) | 1.14 (0.8) | 1.22 (0.3) | 1.12 (1.1) | 1.29 (0.5) | 1.15 (0.7) |
| Week 4 | 0.82 (0.5) | 0.97 (0.6) | 0.98 (0.8) | 1.28 (0.8) | 0.71 (0.4) | 0.87 (0.5) |
| Week 5 | 0.81 (0.5) | 1.08 (0.5) | 0.87 (0.6) | 0.99 (0.7) | 0.78 (0.5) | 1.11 (0.5) |
| Week 6 | 1.04 (0.5) | 1.16 (0.8) | 0.61 (0.6) | 1.47 (1.1) | 1.33 (0.2) | 1.05 (0.7) |
| Week 7 | 1.32 (0.4) | 1.06 (0.7) | 1.32 (0.4) | 0.98 (0.5) | 1.31 (0.5) | 1.09 (0.8) |
| Week 8 | 0.87 (0.5) | 1.26 (0.6) | 1.05 (0.4) | 1.20 (0.4) | 0.76 (0.6) | 1.28 (0.7) |
| Data are presented as average (SD). ^†^ ACWRs are calculated based on registered distance of running event, short-distance (5-10.55km) and long-distance (21.1-42.2km); ^‡^ Running-related knee injury; ^§^ Running-related injury. | | | | | | |
